# Seasonal Influenza Vaccination Coverage Among Women Who Delivered a Live-Born Infant — 21 States and New York City, 2009–10 and 2010–11 Influenza Seasons

**Published:** 2013-12-13

**Authors:** Indu B. Ahluwalia, Helen Ding, Leslie Harrison, Toyia Austin, Denise D’Angelo, Phil Hastings, Nan Ruffo, Mary Elizabeth O’Neil, James A. Singleton, Carolyn B. Bridges

**Affiliations:** Div of Reproductive Health, National Center for Chronic Disease Prevention and Health Promotion; Immunization Svcs Div, National Center for Immunization and Respiratory Diseases, CDC

Because influenza can be especially severe during pregnancy, the American College of Obstetricians and Gynecologists and the Advisory Committee on Immunization Practices recommend influenza vaccination for pregnant women (1,2). Pregnant women experience increased morbidity from influenza infection, and they were at increased risk for severe disease and mortality from 2009 influenza A(H1N1) pdm09 (pH1N1) pandemic virus infection (3–5). During the 2009–10 influenza season, CDC’s Pregnancy Risk Assessment Monitoring System (PRAMS) began collecting data on pregnant women’s vaccination coverage, and 22 areas continued to collect it during the 2010–11 season (6). To estimate state-specific seasonal influenza vaccination coverage among pregnant women for the 2010–11 influenza season, the most recent data available, CDC analyzed data from women who delivered a live-born infant during September 2010–May 2011(N = 18,522). This report describes the results of that analysis, which indicated that, for the 2010–11 season, overall combined 53.6% were vaccinated (44.2% during pregnancy, 8.8% postpartum, and <1% with unknown time during pregnancy). Among those vaccinated during pregnancy, most were vaccinated during the second or third trimester. Wide state-to-state variation in vaccination coverage was observed, with a range of 32.6% to 75.9% and a median of 54.8%. Compared with the 2009–10 season, coverage was either the same or higher in all areas. Strategies that contributed to increased vaccination coverage need to be promoted.

CDC analyzed data from PRAMS,* an ongoing, population-based survey that collects data on a range of maternal behaviors and experiences before, during, and after pregnancy among women who recently delivered a live-born infant. The surveys take stratified random samples of 100–300 women with recent live births monthly from each state birth certificate registry. The selected mothers are mailed up to three questionnaires after delivery; those who do not respond by mail within 2 months are contacted by telephone, and up to 15 attempts are made to reach the women. For the 2010–11 season, 21 states and New York City (NYC) had seasonal influenza vaccination data available.† For this report, CDC analyzed data available on the 2010–11 influenza season from 21 states and NYC among women who had a live birth from September 1, 2010, through May 31, 2011, and responded to PRAMS (N = 18,522). For comparison, vaccination coverage data from the same areas for the 2009–10 season among women who had a live birth from September 1, 2009, through May 31, 2010 (N = 19,429), were also analyzed. The state median response rate was 69.6% (range: 53.7%–85.0%) for the 2009–10 season and 68.2% (range: 55.6%–81.1%) for the 2010–11 season.

Weighted PRAMS data for seasonal vaccination coverage for each of the two seasons were aggregated, and overall estimates of vaccination coverage by area and pregnancy status (pregnant or postpartum) were calculated. To assess the extent and magnitude of changes, the relative percentage point change between two seasons was calculated. Changes in vaccination coverage were reported for each state and NYC, along with information about places where the pregnant women received their vaccination. Women who did not obtain vaccinations were asked to provide reasons why, with the option to select more than one response. All estimates were weighted to adjust for complex survey design and nonresponse.

Seasonal influenza vaccination coverage among women with live births varied among the participating areas, and the median coverage among the states increased from 50.1% during the 2009–10 season to 54.8% in the 2010–11 season (Table 1). All states either maintained or increased their seasonal vaccination coverage from the 2009–10 to the 2010–11 season. Eight (36.4%) of the 22 participating areas reported a statistically significant increase. Areas with the highest percentage increases during the 2010–11 season were Louisiana (from 39.6% to 49.8%), Missouri (from 42.8% to 53.6%), and Washington (from 53.3% to 64.5%).

For the 2010–11 season, the percentage of respondents who reported that their health-care provider recommended vaccination varied by area, ranging from 53.7% to 89.5% (median: 74.3%). Among those who received a provider recommendation or offer of vaccination, median vaccination coverage was 67.1%, ranging from 53.8% in Georgia to 81.9% in Nebraska; among those who did not receive a provider recommendation or offer of vaccination, median vaccination coverage was 18.6%, ranging from 4.0% in Tennessee to 42.4% in Minnesota. Provider recommendation or offer of vaccination was associated with higher influenza vaccination coverage across all areas.

For the 2010–11 season, overall 53.6% of women with live-births reported receiving vaccine, and a majority of these received it during pregnancy (83% [8,715 of 10,533]). Of the women who reported being vaccinated during pregnancy, 4.0% were vaccinated during the first trimester, 17.1% during the second trimester, and 14.4% during the third trimester; the rest were vaccinated during pregnancy, but the trimester could not be ascertained because of missing information. The most common place women reported receiving their influenza vaccination during pregnancy was at their obstetrician/gynecologist’s office (49.3%), followed by the family doctor’s office (14.2%), and work place or school (11.3%) (Table 2). Among women who received an influenza vaccination postpartum, the most common place they reported receiving their vaccination was at the hospital (50.6%), followed by family doctor’s office (15.5%), and their obstetrician/gynecologist’s office (10.5%). Among women who did not receive an influenza vaccination, 71.4% reported the reason was because they “don’t normally get a flu shot,” followed by being “worried about side effect for myself” (53.5%), and “worried that the flu shot might harm my baby” (48.7%) (Table 3). Approximately 41% of nonvaccinated women reported they did not obtain vaccinations because they were “not worried about getting sick from the flu,” and 29% reported they “did not think the flu shot works” (Table 3).

## Editorial Note

Results from this study indicate that historically high seasonal influenza vaccination coverage levels among pregnant women achieved during the 2009–10 season were either maintained or increased during the 2010–11 season by the 21 participating states and NYC (6,7). Influenza vaccination of pregnant women was a focus of public health efforts during the 2009–10 season, with extensive collaborations and mobilization of resources among local, state, federal, and private sector entities. These efforts might have contributed to higher coverage during the 2009–10 season than was observed for previous seasons (1,2,6–8), and might also have contributed to sustained higher rates during the 2010–11 season.

The 2011 American College of Obstetricians and Gynecologist’s recommendations for influenza vaccination of pregnant women and the updated Advisory Committee on Immunization Practices 2010 guidelines, which recommend vaccinations for anyone aged ≥6 months, might lead to further increases in coverage (1–2). As observed during the 2009–10 influenza season, the proportion of respondents who reported that their health-care providers offered or recommended influenza vaccination for 2010–11 varied substantially among states (6). This variation might relate to state-specific approaches to implementing vaccination efforts, differences in health-care delivery infrastructure, or variation in the proportion of pregnant women seeking vaccination. Among those who reported receiving the vaccination during pregnancy, nearly 50% received it from their obstetrician, and those who received it postpartum reported receiving it in the hospital. This information could be useful for guiding vaccination promotion strategies for pregnant and postpartum women.

Variation in vaccination coverage might also relate to differences in state-level policies on vaccine acquisition or distribution and in prevalence or strength of provider offer or recommendation for influenza vaccination in their practices, given that a high correlation was observed between provider recommendation and vaccination. For women who did not report being vaccinated during the 2010–11 season, although the reasons varied overall and by provider recommendation, worries about adverse effects of the influenza vaccine on the woman and her baby, in addition to not getting the flu vaccine as a normative behavior, predominated. In settings where pregnant and postpartum women seek care, continued efforts are needed to encourage providers to recommend and offer influenza vaccination to build on the gains in influenza vaccination coverage made during the 2009–10 and 2010–11 seasons (6–8).

The findings in this report are subject to at least five limitations. First, PRAMS data were available from only 21 states and NYC and might not be generalizable to all women with live births in the United States. For the same 21 states and cohort of pregnant women, PRAMS data compared with internet panel surveys showed similar coverage for the 2010–11 influenza season. Second, the cohort of women available for this analysis, who had live births during September 2010–May 2011, represents only a subset of all women who were pregnant during the influenza seasons. Third, because two influenza vaccines were available during 2009–10 influenza season (seasonal and pH1N1), recall bias might have occurred if women forgot which vaccine they received, leading to potential misclassification of the type of vaccine received. Fourth, because the response rates ranged from 53.7% to 85.0% by state over the two seasons (median: 69.6% for 2009–10 and 68.2% for 2010–11), the findings might be subject to response bias. Finally, mail and telephone respondents might have different demographic characteristics, and women who participated by telephone might have provided responses they perceived to be more socially desirable, although nonresponse analysis and weighting were used to evaluate and adjust for differential response rates between mothers with different characteristics in the PRAMS survey.

What is already known on this topic?

Historically the seasonal vaccination coverage for pregnant women was low, but vaccination rates increased during the 2009–10 season, when vaccination of pregnant women was a focus of public health efforts.

What is added by this report?

Among 21 states and New York City participating in the Pregnancy Risk Assessment Monitoring System, the median proportion of recently pregnant women who reported receiving a seasonal influenza vaccination during the 2010–11 influenza season was 54.8%, compared with 50.1% during the 2009–10 season. All participating states either maintained or increased influenza vaccination coverage among women with live births.

What are the implications for public health practice?

Further efforts are needed that recognize the substantial differences in vaccination rates among geographic areas and the importance of encouraging providers to address pregnant women’s concerns about influenza vaccine safety and effectiveness and to offer influenza vaccination to them.

Based on the findings in this report, seasonal influenza vaccination coverage among women with live-births was higher overall during the 2010–11 influenza season than the 2009–10 season, and estimated coverage was the same as or higher in all 21 participating states and NYC. Despite the gains in coverage from 2009–10 season, 46% of women with live-births did not get vaccinated during the 2010–11 season. Further, among those who reported being vaccinated during pregnancy, a majority of vaccinations occurred during the latter part of pregnancy, which might suggest a need to reinforce messages about the safety of being vaccinated any time during pregnancy. These findings point to the need for continued education of health-care providers and pregnant women regarding the risk for severe illness and pregnancy-related complications from influenza to reduce the burden of influenza on pregnant women and their infants (9,10). These results indicate that providers need to understand the risks and potential barriers to vaccination during pregnancy and develop strategies to address these during encounters with women. Partnerships among various stakeholders at the state, federal, and local levels will be necessary to promote increased implementation of evidence-based vaccination promotion strategies (10).

## Figures and Tables

**TABLE 1:** State-specific seasonal influenza vaccination coverage among women with live births — 21 states and New York City, Pregnancy Risk Assessment Monitoring System, 2009–10 and 2010–11 influenza seasons

State	2009–10 season			2010–11 season			Change between 2009–10 and 2010–11 seasons (%)†
	
	No.	%*	(95% CI)	No.	%*	(95% CI)	
Arkansas	1,055	46.7	(42.6–50.7)	438	46.2	(39.9–52.7)	−1.0
Georgia	614	29.9	(24.3–35.5)	783	32.6	(27.2–38.5)	9.1
Illinois§	1,071	47.1	(43.8–50.3)	1,076	54.1	(50.8–57.3)	14.9
Louisiana§	540	39.6	(34.4–44.8)	662	49.8	(45.1–54.5)	25.7
Maine	709	64.0	(59.9–68.0)	573	63.7	(59.0–68.2)	−0.3
Maryland	1,080	46.1	(41.6–50.7)	1,085	51.8	(47.1–56.5)	12.4
Massachusetts	996	67.5	(63.5–71.4)	1,158	70.9	(67.2–74.4)	5.1
Minnesota§	917	67.9	(64.6–71.1)	848	75.9	(72.6–79.0)	11.8
Missouri§	973	42.8	(39.1–46.6)	932	53.6	(49.8–57.3)	25.1
Nebraska§	1,198	65.4	(62.2–68.5)	901	73.5	(69.9–76.9)	12.5
New Jersey§	1,053	36.8	(33.6–40.0)	1,040	43.6	(40.3–46.9)	18.5
New York	693	54.7	(50.0–59.4)	756	55.5	(50.9–60.0)	1.5
New York City	894	45.9	(41.8–50.0)	985	45.3	(41.5–49.2)	−1.3
Oklahoma	1,432	49.1	(44.6–53.5)	1,221	50.3	(45.5–55.2)	2.6
Rhode Island§	821	63.7	(59.8–67.6)	865	71.7	(68.1–75.1)	12.5
Tennessee	650	41.2	(36.1–46.2)	457	47.2	(41.4–53.0)	14.6
Utah	1,124	57.8	(54.6–61.0)	1,061	57.2	(53.8–60.5)	−1.0
Vermont	742	66.3	(62.8–69.7)	742	65.3	(61.7–68.7)	−1.4
Virginia	318	51.2	(43.9–58.5)	390	58.8	(52.2–65.1)	14.9
Washington§	1,052	53.3	(49.2–57.3)	918	64.5	(60.4–68.3)	21.0
West Virginia	880	44.9	(40.8–48.9)	1,060	49.2	(45.5–53.0)	9.8
Wyoming	617	55.6	(51.0–60.2)	571	55.7	(50.8–60.4)	0.1
*Median*	*945*	*50.1*		*883*	*54.8*		*10.8*
*Minimum*	*318*	*29.9*		*390*	*32.6*		−*1.4*
*Maximum*	*1,432*	*67.9*		*1,221*	*75.9*		*25.7*

**Abbreviation:** CI = confidence interval.

* Weighted to adjust for complex survey design and nonresponse.

† Equals percentage in 2010–11 season minus percentage in 2009–10 season divided by percentage in 2009–10 season multiplied by 100.

§ States that had a statistically significant increase in influenza vaccination coverage in the 2010–11 season compared with the 2009–10 season based on nonoverlapping 95% CIs for the estimates for the two seasons.

**TABLE 2:** Place where influenza vaccination was received among women with live births — 21 states and New York City, Pregnancy Risk Assessment Monitoring System, 2010–11 influenza season

Place of vaccination	During pregnancy			After delivery		
	
	Sample size	%*	(95% CI)	Sample size	%*	(95% CI)
Obstetrician/Gynecologist’s office	4,132	49.3	(47.6–51.0)	198	10.5	(8.6–12.8)
Family doctor or other doctor’s office	1,142	14.2	(13.0–15.4)	332	15.5	(13.2–18.2)
Health department or community clinic	687	8.5	(7.5–9.5)	157	7.2	(5.6–9.2)
Hospital	494	5.7	(5.0–6.5)	937	50.6	(47.0–54.2)
Pharmacy, drug store, or grocery store	628	9.1	(8.2–10.1)	124	8.3	(6.4–10.6)
Work place or school	996	11.3	(10.2–12.4)	90	5.0	(3.7–6.7)
Other place	203	2.1	(1.6–2.6)	64	2.9	(1.9–4.3)
**Total**	**8,282**			**1,902**		

**Abbreviation:** CI = confidence interval.

* Weighted to adjust for complex survey design and nonresponse.

**TABLE 3:** Reasons for not receiving influenza vaccination among women with live births who did not receive an influenza vaccination — 21 states and New York City, Pregnancy Risk Assessment Monitoring System, 2010–11 influenza season

Reason*	Sample size	%†	(95% CI)
Doctor didn’t mention it	6,957	26.7	(25.1–28.4)
Worried about side effect for myself	7,054	53.5	(51.6–55.3)
Worried that the flu shot might harm my baby	7,020	48.7	(46.9–50.6)
Not worried about getting sick from flu	6,910	40.7	(38.8–42.5)
Do not think the flu shot works	6,816	29.4	(27.7–31.1)
Don’t normally get a flu shot	7,117	71.4	(69.7–73.0)
Other reason	5,117	22.1	(20.4–23.9)

**Abbreviation:** CI = confidence interval.

* Women were instructed to select all the applicable reasons they did not receive an influenza vaccination. A total of 7,898 women reported that they were not vaccinated. The sample sizes do not sum to the overall sample size because of missing response information.

† Weighted percentage.

## Footnotes

* Additional information is available at http://www.cdc.gov/prams.

† Questions on the PRAMS influenza supplement included the following: “Since September 2009, did you get a seasonal flu shot? This is different than the H1N1 flu shot.” and “At any time during your most recent pregnancy, did a doctor, nurse, or other health-care worker offer you a seasonal flu shot or tell you to get one?” The question used to assess women’s reasons for not getting flu shot included five items with a yes/no response format, and women could select more than one reason: “What were your reasons for not getting a seasonal flu shot during your most recent pregnancy? For each item, circle Y (yes) if it was a reason for you and N (no) if it was not: 1) My doctor didn’t mention anything about a seasonal flu shot during my pregnancy; 2) I was worried about side effects of the seasonal flu shot for me; 3) I was worried that the seasonal flu shot might harm my baby; 4) I don’t normally get a seasonal flu shot; and 5) other reason—please tell us.”
