# Errata

**Published:** 2014-01-24

## Vol. 62, No. 49

In the report, “Seasonal Influenza Vaccination Coverage Among Women Who Delivered a Live-Born Infant — 21 States and New York City, 2009–10 and 2010–11 Influenza Seasons,” errors occurred in Table 1 on page 1002. For West Virginia, in the three columns for the 2009–10 season, the cells for number, prevalence, and 95% confidence interval should read **1,121, 44.2,** and **(40.6–47.8)**, respectively, and for the last cell in the West Virginia row (“Change between 2009–10 and 2010–11 seasons (%)”), the percentage should read **11.4**. In the row labelled “Median,” in the last cell, the percentage should read **11.6**.

